# A Multicenter Phase II Study on Photodynamic Therapy Using Talaporfin Sodium (ME2906) and a Semiconductor Laser (PNL6405CIN) for Intraepithelial Tumors of the Cervix

**DOI:** 10.1002/rmb2.70001

**Published:** 2025-11-30

**Authors:** Hirotake Murakami, Masaru Sakamoto, Hisamori Kato, Kiyohiko Miyake, Kenji Umayahara, Toshiya Itoh, Madoka Matsuya, Toshiaki Shibata, Toshiyuki Uchida, Masakazu Abe, Tomomi Kotani, Hiroaki Itoh, Naohiro Kanayama

**Affiliations:** ^1^ Department of Obstetrics and Gynecology Hamamatsu University School of Medicine Hamamatsu Japan; ^2^ Department of Gynecology Sasaki Foundation Kyoundo Hospital Tokyo Japan; ^3^ Department of Obstetrics and Gynecology The Jikei University School of Medicine Tokyo Japan; ^4^ Department of Gynecology Kanagawa Preventive Medicine Association Yokohama Japan; ^5^ Department of Gynecology Niizashiki Chuo General Hospital Niiza Japan; ^6^ Department of Nursing, Faculty of Health Science Tokoha University Shizuoka Japan

**Keywords:** fertility preservation, photochemotherapy, photosensitizing agents, porphyrins uterine cervical dysplasia

## Abstract

**Purpose:**

Conization for high‐grade cervical intraepithelial neoplasia (CIN2–3) often harms obstetric outcomes. Photodynamic therapy (PDT) with talaporfin sodium provides a cervix‐sparing alternative with short photosensitivity. We investigated the efficacy and safety of talaporfin‐PDT for CIN2–3.

**Methods:**

We conducted a prospective, multicenter, single‐arm phase II trial (jRCT2041190087) in women ≥ 20 years with biopsy‐proven CIN2–3. Patients received talaporfin sodium (40 mg/m^2^) and 664‐nm laser irradiation. Efficacy was evaluated by cytology/histology from weeks 12 to 24; the primary endpoint was complete response (CR) versus an 85% threshold. Safety, HPV status, cervical length, and reproductive outcomes were monitored.

**Results:**

Of 88 enrolled, 79 were treated and 77 (CIN2 = 7; CIN3 = 70; median age 32 years; 93.5% desiring future pregnancy) were evaluable. CR was achieved in all 77 (100%; lower 95% CI: 96.2%), including 95.8% in CIN3. High‐risk HPV clearance occurred in 82.4%. One recurrence (1.3%) was observed. No serious adverse events occurred; four grade ≥ 3 events resolved. Cervical length was preserved. Eleven pregnancies occurred, yielding eight full‐term deliveries.

**Conclusions:**

Talaporfin‐PDT showed excellent efficacy, safety, and fertility preservation in CIN2–3, supporting its potential as a non‐excisional alternative.

**Trial Registration:**

This study was registered in the Japan Registry of Clinical Trials (jRCT) under the identifier jRCT2041190087

## Graphical abstract

Talaporfin sodium‐PDT showed 100% complete response, preserved cervical length, cleared high‐risk HPV in 82.4%, had minimal recurrence, no serious safety issues, and enabled successful full‐term pregnancies, indicating effective and fertility‐sparing treatment for CIN2–3.

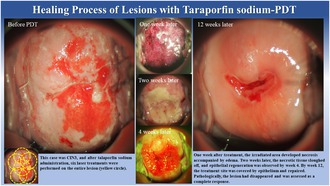

## 1 Background

Cervical cancer is a tumor related to HPV infection, which may be eradicated through widespread vaccination and appropriate cancer screening and treatment. Although HPV vaccination has been conducted in many countries and a gradual decrease in the number of cervical cancer cases has been reported [1], cervical cancer remains an important disease that threatens the lives of and reproduction of the young generation worldwide. Cervical intraepithelial lesions (CIN) include low‐grade intraepithelial lesions (LSIL), such as mild squamous dysplasia (CIN1), and high‐grade intraepithelial lesions (HSIL) such as moderate squamous dysplasia; CIN2, severe squamous dysplasia and carcinoma in situ; CIN3. LSIL often resolve spontaneously without intervention and are regularly followed up. On the other hand, HSIL require treatment because some may progress to advanced cancer.

Conization is a recommended treatment for HSIL because of its reliable diagnosis and cure rate. The impact of conization on reproductive and obstetric outcomes is an increased risk of miscarriage, preterm delivery, and an increased rate of Cesarean section [2, 3]. In developed countries, the number of patients who require treatment for CIN before conception is increasing [4]. A recent study from Japan showed a high rate of preterm delivery after conization (25.3%) [5]. Preterm delivery leads to a low birth weight, which is associated with respiratory and neurological complications, as well as long‐term sequelae, such as metabolic syndrome and mortality [6, 7]. The cervix must be preserved because the cervical length after conization is related to the risk of preterm birth [8, 9, 10]. Laser vaporization and photodynamic therapy (PDT) are the most common methods of treatment. Laser vaporization was previously shown to be less effective than conization for CIN3, and PDT for CIN was initiated in Japan in 1996 using porfimer sodium, a first‐generation photosensitizing agent, and excimer dye laser with good outcomes. However, due to the long excretion time and the occurrence of photosensitivity reactions, PDT using porfimer sodium for CIN3 was not recommended as a standard treatment. On the other hand, talaporfin sodium, a second‐generation photosensitizer developed in Japan, is characterized by a short excretion time. PDT using talaporfin sodium and a semiconductor laser was previously shown to be effective and safe for the treatment of early lung cancer [11], recurrent esophageal cancer after chemoradiation therapy, and brain tumors [12, 13], whereas its effects on CIN currently remain unknown.

Therefore, we herein conducted a single‐arm prospective phase II study to examine the efficacy and safety of PDT using talaporfin sodium for CIN.

## 2 Methods

### 2.1 Trial Design

This was a prospective, multicenter, non‐randomized, open‐label, single‐arm phase II study based at Hamamatsu University Hospital (approval no. 710). The study was conducted in accordance with Good Clinical Practice Guidelines for Drugs and Medical Devices and the Declaration of Helsinki (jRCT2041190087).

**FIGURE 1:**
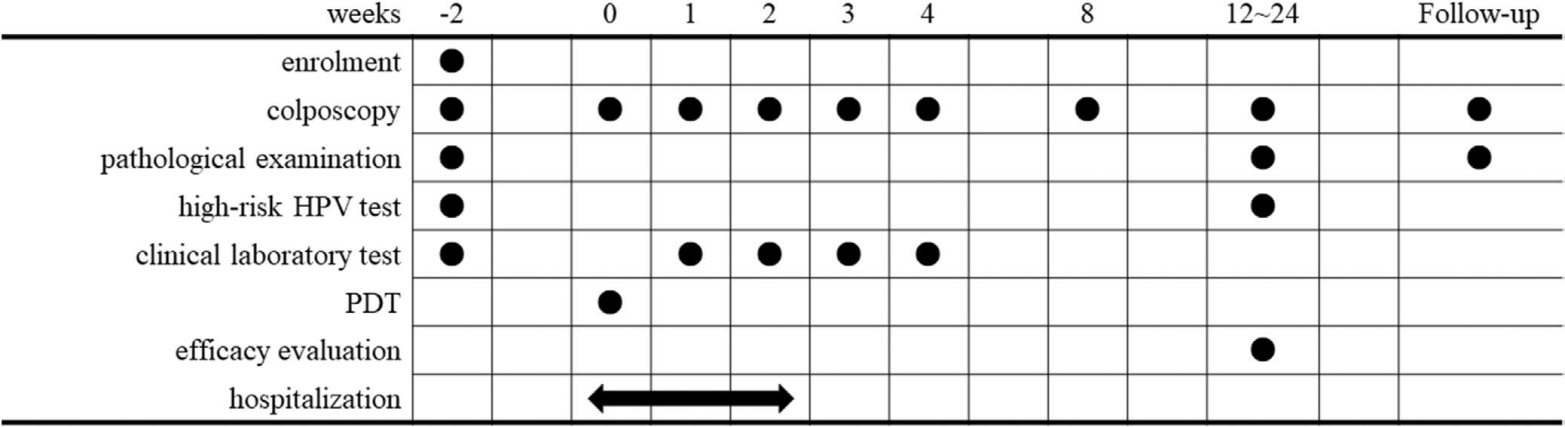
Overview of the protocol. This diagram shows the sequence of study procedures: Eligibility assessment, intervention implementation, and follow‐up visits. Assessment timelines for clinical and laboratory outcomes and an efficacy evaluation are shown.

The study protocol is shown in Figure 1.

#### 2.1.1 Study Population

The number of patients needed to reach a significant difference was calculated as 68, and in consideration of a drop‐out rate of 10%, the final number of patients required was 75.

### 2.2 Inclusion Criteria

1. Female patients of at least 20 years of age at the time of consent.
2. Patients diagnosed with CIN2 or CIN3 by cervical cytology and punch biopsy under colposcopy.
3. Patients not seeking any treatment other than PDT for CIN.
4. An Eastern Cooperative Oncology Group performance status of 0–1
5. Patients with all of the following screening test results and adequate functions in major organs: white blood cell count ≥ 3000/mm^3^, platelet count ≥ 100000/mm^3^, AST and ALT ≤ twice the upper institutional limit, serum total bilirubin ≤ 2.0 mg/dL, and BUN and serum creatinine ≤ 2.0 mg/dL, being ≤ 1.5 × the upper limit of the institutional reference value.
6. Patients who provided their written consent to participate in the clinical trial.

### 2.3 Exclusion Criteria

1. Patients with adenodysplasia, intraepithelial adenocarcinoma, squamous cell carcinoma, or other cervical malignancies.
2. Patients with Unsatisfactory Colposcopic Findings (UCF) or transformation zone type 3 (TZtype 3) with deep cervical involvement by colposcopy
3. Patients with multiple cancers
4. Patients with poorly controlled cardiac, respiratory, hepatic, renal, gastrointestinal, hematological, endocrine, neurological, or psychiatric diseases
5. Patients with pre‐existing or concomitant photosensitivity
6. Patients with porphyria
7. Patients previously treated with PDT using talaporfin sodium or porfimer sodium
8. Pregnant or possibly pregnant women, lactating women, and female patients who wished to become pregnant by the end of the observation period of this study (maximum 24 weeks after PDT treatment)
9. Patients unable to use an adequate method of contraception or obtain adequate contraceptive consent from the time of obtaining consent until the completion of the observation period of this study
10. Patients participating in another clinical trial or had completed participation within at least 3 months.
11. Patients judged by the investigator or subinvestigator to be inappropriate for this clinical trial.

### 2.4 Treatment Procedures

#### 2.4.1 PDT Treatment

**FIGURE 2:**
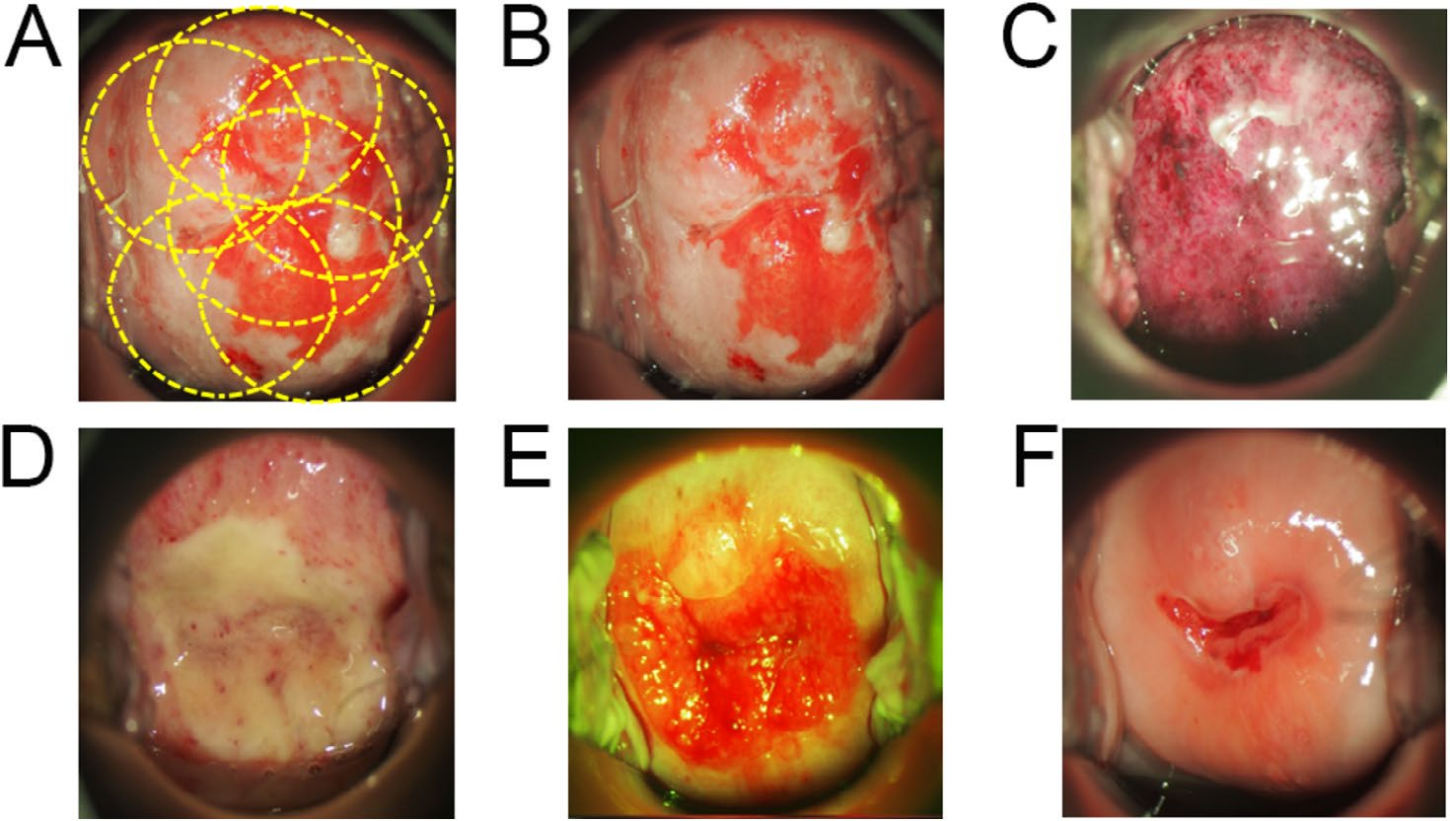
Representative treatment course of CIN3 lesion treated with talaporfin‐PDT. (A) After talaporfin sodium administration, six laser illuminations were delivered to cover the entire lesion (yellow circle). (B–F) Cervical findings before talaporfin‐PDT (B) and at 1 (C), 2 (D), 4 (E), and 12 (F) weeks after treatment.

The PDT procedure consisted of the intravenous administration of 40 mg/m^2^ of talaporfin sodium and laser irradiation with a 664 nm semiconductor laser 4–6 h after drug administration. The distance between the lesion and probe tip was maintained so that the area treated by single laser irradiation of the vaginal lesion was 20 mm in diameter. Regarding uterovaginal lesions, lesion extent was delineated by colposcopy. Laser irradiation was delivered in one or more passes to ensure complete coverage of the lesion, including several millimeters of the adjacent normal epithelium beyond the lesion margins (Figure 2A), as recommended in previous PDT protocols for CIN [14]. Figure 2B–F presents a representative course of treatment. Laser irradiation of the cervix was performed to a depth of 20 mm from the external uterine opening. In both uterovaginal and intracervical laser irradiation, irradiation power density was 150 mW/cm^2^ and irradiation energy density was 100 J/cm^2^.

#### 2.4.2 Clinical Examination Items

Samples were collected as part of the safety evaluation before treatment, 3 days after treatment, and weekly until 4 weeks after treatment. The following laboratory parameters were examined: the red blood cell count, hemoglobin, white blood cell count, white blood cell fraction, platelet count, PT‐INR, total protein, albumin, total bilirubin, AST, ALT, γ‐GTP, ALP, LDH, BUN, creatinine, Na, K, Cl, CRP, Ca, urine protein, urine sugar, and urine urobilinogen.

#### 2.4.3 Toxicity Assessment

Regarding safety, adverse events were defined as new events that occurred between the administration of the study drug and 4 weeks after PDT (treatment period). Adverse events were graded for severity according to CTCAE ver. 5.0. All serious adverse events were immediately reported to the Efficacy and Safety Committee for evaluation.

#### 2.4.4 Follow‐Up and Evaluation

All patients were kept out of direct sunlight from the time of drug administration and remained in rooms and areas of the facility with light levels < 500 Lux for at least 2 weeks. They were then discharged. Patients were evaluated weekly by a physical examination, the measurement of hematological and biochemical variables in blood, and a post‐treatment follow‐up by colposcopy until 28 days after PDT. Eight weeks after the PDT treatment, colposcopy and a physical examination were performed to confirm the absence of uterine orifice closure or stricture. Cervical cytology and histology were used to assess treatment efficacy every 4 weeks from 12 to 24 weeks after treatment. The treatment response assessment was repeated until a treatment response was confirmed, the observation period was terminated, and patients were followed up when CR was confirmed or when residual lesions were detected at 24 weeks. Cervical length and HPV tests were performed at 12 weeks and at the time of the treatment response assessment, and the results obtained were compared with those at the time of enrollment. The follow‐up with cervical cytology and colposcopy was conducted every 12 weeks during the follow‐up period. Pregnancy was allowed 24 weeks post‐treatment in patients with a desire to have a baby. The minimum follow‐up period was 56 weeks after treatment, and every 12 weeks after 56 weeks until the end of the study period.

#### 2.4.5 Efficacy Assessment Procedure

Patients underwent cervical cytology, cervical histology, and colposcopic imaging every 4 weeks from 12 weeks after treatment until treatment efficacy was confirmed. Patients were classified into four groups based on the findings of cervical cytology and colposcopy: a complete response (CR), progressive disease (PD), non‐CR/non‐PD, and not evaluable (NE). Criteria for CR were based on the algorithm for negative findings in both cervical cytology and histology (Table S1). Cervical cytology with NILM, ASC‐US, or LSIL was considered to be negative. No abnormal findings (NAF) or LSIL were considered to be negative in cervical histology. CR was defined as the absence of HSIL (CIN2–3) on follow‐up histopathology and was often used to assess the efficacy of laser vaporization [15, 16]. Cytologic findings of ASC‐US or LSIL, which are generally managed with surveillance rather than treatment, were therefore classified as CR in this study. When cytology and histology were both negative, the overall diagnosis was CR. If cervical cytology or histology showed worsening of the lesion from that before treatment with the study drug, the patient was considered to have PD. Patients were classified as NE if it was not possible to have a pathological examination for any reason. Pre‐ and post‐treatment colposcopy and pathology decisions were made by one pathologist at a central pathology center independent of the four centers where this study was conducted, and the diagnosis of all specimens was confirmed by one pathologist. In addition, the overall pre‐ and post‐treatment evaluation, including the validity of colposcopy images and biopsy sites at the time of both the pre‐treatment diagnosis and treatment efficacy assessment and the results of the central pathology assessment, was performed for all cases by an independent central determination committee consisting of two experienced gynecological oncologists. The final pre‐treatment diagnosis and efficacy results were selected by this committee.

### 2.5 Endpoint

#### 2.5.1 Efficacy

Primary endpoint: The CR to pretreatment lesion ratio.

Secondary endpoints: The HPV‐negative rate, recurrence rate and duration of recurrence, and the rate of preterm births among deliveries during the follow‐up period.

#### 2.5.2 Safety Assessment

The incidence of adverse events, laboratory test results, and skin photosensitivity test results.

### 2.6 Statistical Analysis

In the present study, the threshold for the CR rate was set at 85%, using the 84.8% negative resection margin rate for conical resection for CIN3 as an external control, based on a 2018 study showing the findings of a nationwide survey in Japan [17]. Frequency summaries of the best response to treatment were performed for the CR rate and its 95% confidence interval (Clopper‐Pearson exact confidence interval). Regarding the primary endpoint of the CR rate, treatment was considered to be effective if the lower limit of the 95% confidence interval exceeded the 85% threshold in CIN3 cases. The Kaplan–Meier method was used to calculate appropriate summary statistics, such as the annual percentage and median, for overall survival (OS) and the duration of recurrence. The preterm birth rate was calculated as the 95% confidence interval for the percentage of cases delivered preterm at < 37 weeks using the denominator of cases that were conceived and delivered during the follow‐up period. Changes in cervical length were compared based on transvaginal ultrasound measurements by a paired‐*t* test before and after PDT. All authors had access to the study data and reviewed and approved the final manuscript.

## 3 Results

### 3.1 Patients

**FIGURE 3:**
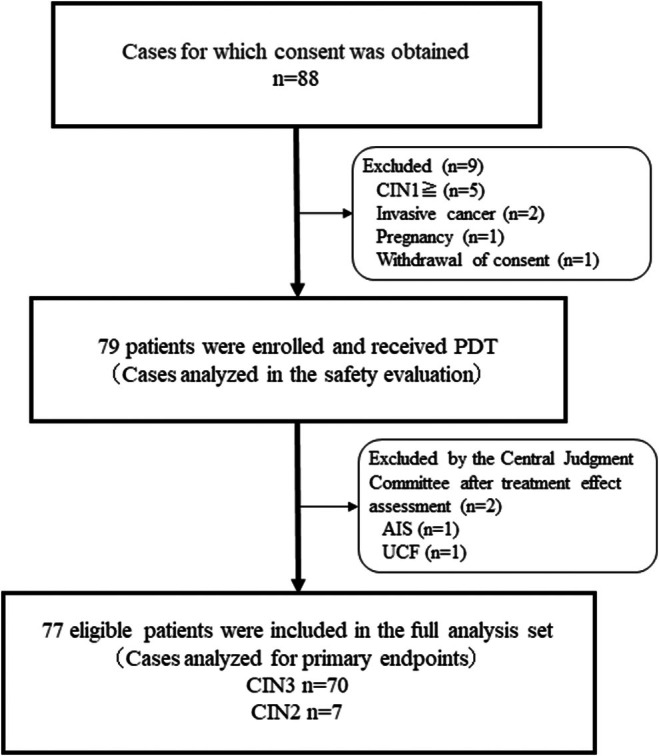
Flow chart of participants. The diagram outlines the selection of participants, including initial enrollment and reasons for exclusion. The final sample size for the safety evaluation (*n* = 79) and full analysis (*n* = 77) are indicated.

Consent was obtained for 88 patients at four centers between February 2020 and February 2023, and 79 patients who met the inclusion criteria received PDT. Seventy‐seven patients (CIN2: 7, CIN3: 70), except for 2 who were excluded from the analysis by the Central Judgment Committee due to “UCF by colposcopy” and “concurrent intraepithelial adenocarcinoma,” were included in the efficacy analysis population (Figure 3), while the safety analysis population included all 79 patients who received PDT.

The characteristics of the study population are shown in Table 1. Median age at consent was 32.0 years (range 24–63 years) for 77 patients; 23 (29.9%) were in their 20s, and 48 (62.3%) were in their 30s. Sixty‐three women (81.8%) had zero deliveries, 14 (18.2%) had one or more deliveries, and 72 (93.5%) wanted to have a baby. Three patients had received treatment prior to study participation: one by endocervical conization and two by laser transpiration. Five (7.1%) of the 77 patients had a history of HPV vaccination. There were 74 pre‐treatment high‐risk HPV‐positive cases (96.1%).

**TABLE 1:** Characteristics of patients.

	CIN3	CIN2	Total
Number of patients	70	7	77
Age			
20 ~ 29 years	20 (28.6)	3 (42.9)	23 (29.9)
30 ~ 39 years	44 (62.9)	4 (57.1)	48 (62.3)
40 ~ 49 years	5 (7.1)	0 (0.0)	5 (6.5)
50 ~ 59 years	0 (0.0)	0 (0.0)	0 (0.0)
≦ 60 years	1 (1.4)	0 (0.0)	1 (1.3)
Parity			
0	57 (81.4)	6 (85.7)	63 (81.8)
1 and more	13 (18.6)	1 (14.3)	14 (18.2)
Previous treatment			
Conization	1 (1.4)	0 (0.0)	1 (1.3)
Laser vaporization	2 (2.9)	0 (0.0)	2 (2.6)
HPV vaccination	4 (5.7)	1 (14.3)	5 (6.5)
Cervical cytology			
ASC‐H	5 (7.1)	3 (42.9)	8 (10.4)
HSIL	65 (92.9)	4 (57.1)	69 (89.6)
HR‐HPV detection			
Positive	67 (95.7)	7 (100.0)	74 (96.1)
Negative	3 (4.3)	0 (0.0)	3 (3.9)

*Note:* Variables are represented as *n* (%).

Abbreviations: ASC‐H, atypical squamous cells cannot exclude high‐grade squamous intraepithelial lesion; HPV, human papillomavirus; HR‐HPV, high risk human papillomavirus; HSIL, high‐grade squamous intraepithelial lesion.

### 3.2 Efficacy

All 77 patients achieved CR by the time of the efficacy evaluation, and the lower limit of the 95% confidence interval was 96.2% (95.8% for CIN3 only). The results of the pathological diagnosis at the time of CR were negative in both cytology and histology for 71 patients (92.2%). In the CIN3 group (*n* = 70), the histology of ASC‐US (*n* = 4, 5.7%) was identified as NAF (*n* = 3, 4.3%) and CIN1 (*n* = 1, 1.4%). Of the 2 patients with LSIL (2.9%), 1 had NAF and the other had CIN1 (1.4% each) (Table 2).

**TABLE 2:** Results of pathological diagnosis when determining CR.

Cytology	Histology	Comprehensive diagnosis	CIN3	CIN2	Total
NILM	NAF	Negative	64 (91.4)	7 (100.0)	71 (92.2)
ASC‐US	NAF	LSIL	3 (4.3)	0 (0.0)	3 (3.9)
	CIN1	LSIL	1 (1.4)	0 (0.0)	1 (1.3)
LSIL	NAF	LSIL	1 (1.4)	0 (0.0)	1 (1.3)
	CIN1	LSIL	1 (1.4)	0 (0.0)	1 (1.3)

*Note:* Variables are represented as *n* (%).

Abbreviations: ASC‐US, atypical squamous cells of undetermined significance; CIN; cervical intraepithelial neoplasia; CR, complete response; LSIL, low‐grade squamous intraepithelial lesion; NAF, no abnormal findings; NILM, negative for intraepithelial lesion or malignancy.

Of the 74 patients who were positive for high‐risk HPV before treatment, 61 (82.4%) were negative at the time of the treatment response assessment; 55 (82.1%) of 67 HPV‐positive patients in the CIN3 group and 6 (85.7%) of 7 HPV‐positive patients in the CIN2 group were negative (Table S2). The percentage of pretreatment HPV types, including duplicates, was the highest for type 16 (41.6%) in 32 patients, with 35.1% for type 52, 19.5% for type 58, 16.9% for type 31, and 11.7% for type 18.

CIN3 recurrence was observed in one patient (1.3%) after they were transferred to the follow‐up period; it was detected 204 days after the PDT treatment, was considered to be recurrence, and conization was performed. None of the other 76 patients had recurrence during the follow‐up period of 384–1083 days. Furthermore, no patient died within the study period, and the OS rate was 100%.

**FIGURE 4:**
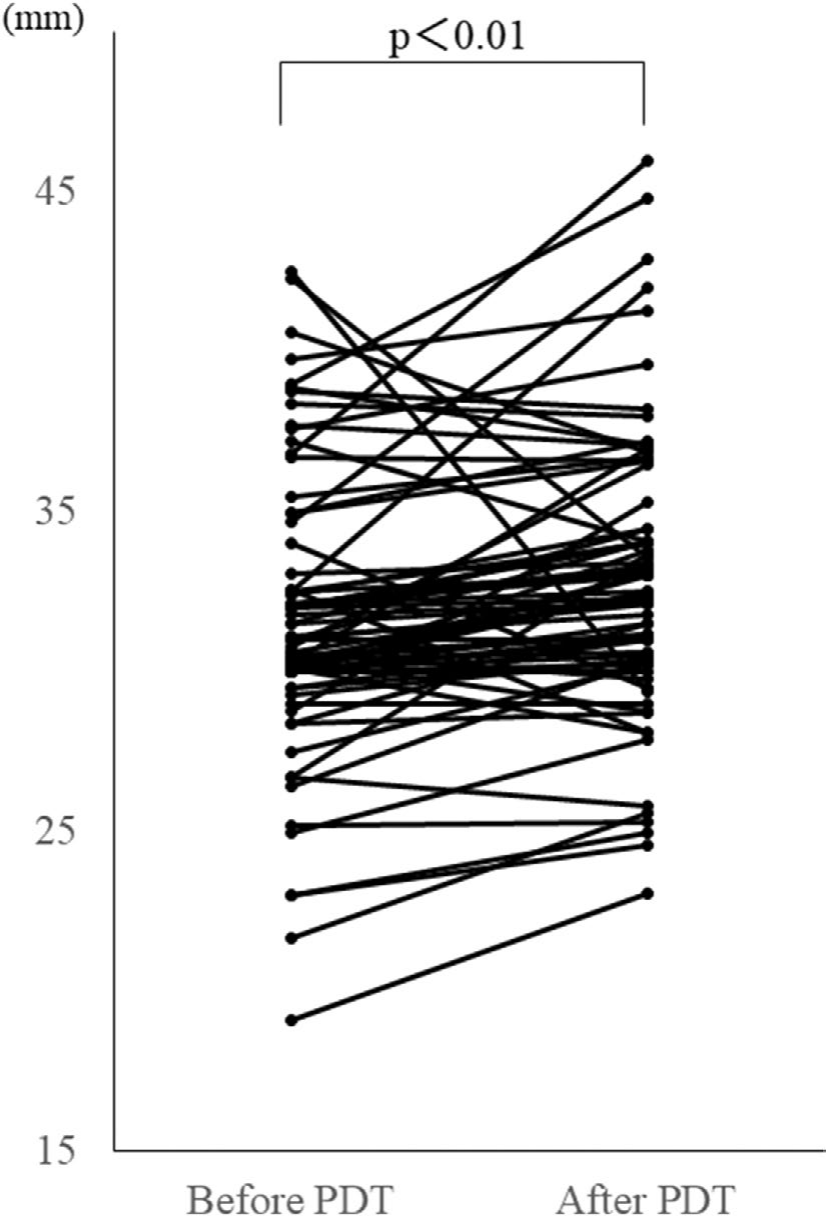
Comparison of cervical lengths before and after PDT. Cervical lengths (mm) were compared before and after PDT by a paired *t*‐test.

During the follow‐up, 11 patients (14.3%) became pregnant, 8 had full‐term deliveries by the endpoint, and four were diagnosed with impending preterm labor and were treated with oral medication. The median length of the cervix was 31.6 mm before treatment and 32.8 mm at the time of the treatment effect assessment (*p* < 0.01), indicating no shortening of the cervix and an increased likelihood of elongation (Figure 4).

### 3.3 Adverse Events

Common adverse events that occurred in more than 10% of patients in this clinical study are shown in Table 3. Grade 3 or higher adverse events occurred in four patients, and all recovered during the follow‐up period. Two of the Grade 3 or higher adverse events, namely, elevated ALT in one case and elevated γ‐GTP in the other, were causally related to the treatment.

**TABLE 3:** Major adverse events by PDT.

	Total	Grade 1	Grade 2	Grade 3
Lower abdominal pain	68 (88.3)	4 (5.2)	64 (83.1)	0 (0.0)
Hypoalbuminemia	52 (67.5)	52 (67.5)	0 (0.0)	0 (0.0)
Blood cell disorders				
Anemia	15 (19.5)	13 (16.9)	2 (2.6)	0 (0.0)
Neutropenia	1 (1.3)	0 (0.0)	0 (0.0)	1 (1.3)
Dysmenorrhea	14 (18.2)	1 (1.3)	13 (16.9)	0 (0.0)
Clinical examination				
Increased γGTP	13 (16.9)	8 (10.4)	4 (5.2)	1 (1.3)
ALT increase	8 (10.1)	5 (6.5)	2 (2.6)	1 (1.3)
Insomnia	13 (16.9)	2 (2.6)	11 (14.3)	0 (0.0)
Photosensitivity	13 (16.9)	13 (16.9)	0 (0.0)	0 (0.0)
Fever	12 (15.6)	11 (14.3)	1 (1.3)	0 (0.0)
Headache	11 (14.3)	7 (9.1)	4 (5.2)	0 (0.0)
Proteinuria	10 (13.0)	8 (10.4)	2 (2.6)	0 (0.0)
Nausea	10 (13.0)	8 (10.4)	2 (2.6)	0 (0.0)
Diarrhea	10 (13.0)	9 (11.7)	0 (0.0)	1 (1.3)

*Note:* Only adverse events with a frequency of 10% or greater are listed among all patients (*n* = 77). Variables are represented as *n* (%). Grade 4–5 was not observed.

In the 79 patients treated with the drug, there were 400 adverse events of any cause, of which 194 occurred in 72 patients (91.1%) and were causally related to PDT. There were no serious adverse events or adverse events leading to death.

The most common adverse event with a causal relationship to talaporfin sodium PDT was lower abdominal pain in 68 patients (86.1%), 64 (94.1%) of whom with Grade 2 pain were treated with some form of analgesia. The second most common adverse event was hypoalbuminemia in 52 patients (65.8%), all of whom were Grade 1 and resolved spontaneously.

## 4 Discussion

In the present study, 70 patients were treated with talaporfin sodium PDT for CIN3. The CR rate was 100%, and the lower limit of the 95% confidence interval was 95.8%, exceeding the threshold value of 85%. Among the 8 patients that progressed to delivery during the follow‐up period after CR confirmation, no preterm births were observed. In addition, there were no serious adverse events. Although there were four Grade 3 or higher adverse events, all patients showed improvement, suggesting the safety of this treatment.

The CR rate for talaporfin sodium PDT was 100%. Among CR cases, those diagnosed as LSIL at the time of the treatment efficacy assessment became negative during the follow‐up period. This result was superior to the 82.9% CR rate achieved with talaporfin sodium PDT for lung squamous cell carcinoma [11]. CR rates by PDT using various photosensitizers for CIN in previous studies were as follows: porfimer sodium PDT, 90%–100% [18, 19]; 5‐ALA PDT, 31.3%–98.2% [20, 21, 22]; Hexaminolevulinate PDT, 62.5% [22]; Methylaminolevulinate PDT, 90% [23]; Photolon PDT, 92.9% [24]. Although different photosensitizers were utilized, the PDT cure rate was similar to the negative margin rate of 84.8% [17] achieved with cone biopsy. Among these photosensitizers, Photolon possesses a chlorin ring, similar to talaporfin sodium. Efficacy in the present study was equal to or greater than that of Photolon, suggesting that talaporfin sodium PDT is effective for the treatment of CIN.

Laser vaporization is a cervix‐preserving treatment for CIN with established efficacy. In cohorts monitored with scheduled histopathologic follow‐up, Suzuki et al. reported recurrence rates of 3.5% at 1 year and 5.2% at 2 years [16], whereas Shimada et al. reported 5.1% at 1 year, 6.4% at 2 years, and 9.5% at 5 years [15]. Recurrence was defined as detection of HSIL or worse on follow‐up pathology, with the definition used in this study. For CIN3, one‐year CR rates estimated from recurrence ranged from 77.4% to 96.5% across reports [25]. In the present study, talaporfin sodium PDT achieved a CR of 100%, with a recurrence rate of 1.4% at a median follow‐up of 1.5 years, indicating efficacy comparable to or possibly exceeding that of laser vaporization.

Furthermore, although talaporfin sodium PDT had a protocol‐dependent 2‐week hospital stay, the average hospital stay for esophageal cancer and brain tumors is 7–10 days. Post‐discharge lifestyle guidance, such as light shielding, will reduce the length of hospitalization further. Since the average hospital stay for porfimer sodium is 3–4 weeks, talaporfin sodium PDT is useful from a medical economic standpoint.

The HPV‐negative conversion rate with talaporfin sodium PDT was 82.4%. Reported HPV‐negative conversion rates for PDT with various agents were as follows: porfimer sodium PDT, 73%–80.6% [18, 19]; 5‐ALA PDT, 83.9%–90.9% [21, 26]; Hexaminolevulinate PDT, 62.5% [27]; Photolon PDT, 53.4% [24]. The present study confirmed a high HPV‐negative rate, which may have contributed to the low recurrence rate of 1.4% during the follow‐up period. There was only one case of recurrence, and that patient had persistent HPV 68 infection after PDT treatment. Persistent HPV infection is a well‐established risk factor for recurrence, even after cervical conization. In this study, 17.6% of patients had persistent HPV after PDT; these patients are undergoing long‐term follow‐up, and long‐term outcomes will be reported in the future. PDT has been reported to exert an immunostimulatory effect [28], and the high HPV‐negative rate in the present study may be a secondary effect of PDT, which is not observed with conization.

There were 11 pregnancies during the follow‐up in the present study, and all 8 deliveries were full‐term births. The rate of preterm delivery in pregnancies after conization is elevated and has been an issue in the standard treatment of CIN cases of reproductive age. In this study, we confirmed a change in cervical length before and after PDT and found no shortening of the cervical canal. A significant increase in cervical length was detected after PDT; however, the magnitude of the increase was small (31.6 vs. 32.8 mm) and, thus, was not considered to be clinically significant. Although there were only 8 deliveries in the present study, all were full‐term, suggesting that PDT reduced the adverse event of preterm delivery. Conization causes shortening of cervical length which is directly related to preterm delivery. On the other hand, this PDT does not shorten the cervical canal and, thus, may reduce preterm prematurity in CIN3 patients more than standard treatment. This PDT may provide a new treatment option for women with CIN3 who wish to have a baby.

There are several limitations that need to be addressed. This was a single‐arm study and was not a comparison of efficacy with conization, the standard of care. Therefore, the Ethics Committee pointed out that since PDT is a treatment for cervical preservation, it was not ethical to conduct a randomized trial on PDT versus conization, which increases the rate of preterm delivery. Based on this decision, the present study was performed as a single‐arm trial using external subjects to confirm the efficacy of the procedure, for which a large study population is required. Furthermore, the minimum follow‐up period in this study was 56 weeks after treatment, which may have been too short because CIN3 has been shown to recur a long period of time after treatment. Therefore, the short follow‐up period of 54–154 weeks in the present study may also be an issue. In addition, the number of deliveries during the follow‐up period was small (*n* = 8). Although this was not a sufficient number of cases to evaluate the preterm delivery rate, the long follow‐up period needed to obtain a sufficient number of cases was not available during the present study to examine efficacy and safety. However, no shortening in cervical length was observed. The high risk of preterm birth has been related to cervical shortening. Therefore, the result showing no shortening strongly supports the potential for reducing the risk of preterm birth. We will continue to follow the patients in this study and accumulate more data.

## 5 Conclusions

PDT using talaporfin sodium and a semiconductor laser achieved a high cure rate and HPV negativity for CIN. No serious adverse events or shortening of the cervical length were observed, suggesting the potential of PDT to prevent preterm delivery. These results indicate that PDT is an effective treatment option for CIN patients who wish to have a baby.

## Funding

This study was financially supported by Meiji Seika Pharma Co. Ltd. (to Hirotake Murakami). The sponsor had no role in the design, conduct, data interpretation, or publication of this study.

## Ethics Statement

This study was approved by the Ethics Committee of Hamamatsu University School of Medicine (Approval No. 710). All procedures followed were in accordance with the ethical standards of the responsible committee on human experimentation (institutional and national) and with the Helsinki Declaration of 1964 and its later amendments.

## Consent

Written informed consent was obtained from all patients for being included in the study.

## Conflicts of Interest

Hirotake Murakami has received research funding from Meiji Seika Pharma Co. Ltd., as above mentioned. The other authors declare that they have no competing interests.

## Supporting information


**Data S1:** rmb270001‐sup‐0001‐supinfo.docx.

## Data Availability

The datasets generated and/or analyzed during the current study are not publicly available due to restrictions based on the informed consent process and the requirement to protect participant privacy. De‐identified data may be available from the corresponding author on reasonable request and with permission of the Ethics Committee of Hamamatsu University School of Medicine.

## References

[1] J. Lei , A. Ploner , K. M. Elfstrom , et al., “HPV Vaccination and the Risk of Invasive Cervical Cancer,” New England Journal of Medicine 383, no. 14 (2020): 1340–1348. 32997908 10.1056/NEJMoa1917338

[2] M. Kyrgiou , G. Koliopoulos , P. Martin‐Hirsch , M. Arbyn , W. Prendiville , and E. Paraskevaidis , “Obstetric Outcomes After Conservative Treatment for Intraepithelial or Early Invasive Cervical Lesions: Systematic Review and Meta‐Analysis,” Lancet 367, no. 9509 (2006): 489–498. 16473126 10.1016/S0140-6736(06)68181-6

[3] M. Kyrgiou , A. Mitra , M. Arbyn , et al., “Fertility and Early Pregnancy Outcomes After Treatment for Cervical Intraepithelial Neoplasia: Systematic Review and Meta‐Analysis,” BMJ (Clinical Research Ed.) 349 (2014): g6192. 10.1136/bmj.g6192 PMC4212006 25352501

[4] J. Ting , D. T. Kruzikas , and J. S. Smith , “A Global Review of Age‐Specific and Overall Prevalence of Cervical Lesions,” International Journal of Gynecological Cancer 20, no. 7 (2010): 1244–1249. 21495248 10.1111/igc.0b013e3181f16c5f

[5] K. Miyakoshi , A. Itakura , T. Abe , et al., “Risk of Preterm Birth After the Excisional Surgery for Cervical Lesions: A Propensity‐Score Matching Study in Japan,” Journal of Maternal‐Fetal & Neonatal Medicine 34, no. 6 (2021): 845–851. 31092078 10.1080/14767058.2019.1619687

[6] A. M. Ahmed , S. M. Grandi , E. Pullenayegum , et al., “Short‐Term and Long‐Term Mortality Risk After Preterm Birth,” JAMA Network Open 7, no. 11 (2024): e2445871. 39565625 10.1001/jamanetworkopen.2024.45871 PMC11579792

[7] C. Amadou , P. Y. Ancel , J. Zeitlin , C. Ribet , M. Zins , and M. A. Charles , “Long‐Term Health in Individuals Born Preterm or With Low Birth Weight: A Cohort Study,” Pediatric Research 97, no. 2 (2025): 577–585. 38965395 10.1038/s41390-024-03346-6 PMC12015107

[8] S. V. Firichenko , M. Stark , and O. A. Mynbaev , “The Impact of Cervical Conization Size With Subsequent Cervical Length Changes on Preterm Birth Rates in Asymptomatic Singleton Pregnancies,” Scientific Reports 11, no. 1 (2021): 19703. 34611206 10.1038/s41598-021-99185-0 PMC8492699

[9] M. Kacerovsky , I. Musilova , S. Baresova , et al., “Cervical Excisional Treatment Increases the Risk of Intraamniotic Infection in Subsequent Pregnancy Complicated by Preterm Prelabor Rupture of Membranes,” American Journal of Obstetrics and Gynecology 229, no. 1 (2023): e1–e13. 10.1016/j.ajog.2022.12.316 36596440

[10] M. Kyrgiou , A. Athanasiou , I. E. J. Kalliala , et al., “Obstetric Outcomes After Conservative Treatment for Cervical Intraepithelial Lesions and Early Invasive Disease,” Cochrane Database of Systematic Reviews 11, no. 11 (2017): Cd012847. 29095502 10.1002/14651858.CD012847 PMC6486192

[11] H. Kato , K. Furukawa , M. Sato , et al., “Phase II Clinical Study of Photodynamic Therapy Using Mono‐L‐Aspartyl Chlorin e6 and Diode Laser for Early Superficial Squamous Cell Carcinoma of the Lung,” Lung Cancer 42, no. 1 (2003): 103–111. 14512194 10.1016/s0169-5002(03)00242-3

[12] T. Yano , H. Kasai , T. Horimatsu , et al., “A Multicenter Phase II Study of Salvage Photodynamic Therapy Using Talaporfin Sodium (ME2906) and a Diode Laser (PNL6405EPG) for Local Failure After Chemoradiotherapy or Radiotherapy for Esophageal Cancer,” Oncotarget 8, no. 13 (2017): 22135–22144. 28212527 10.18632/oncotarget.14029 PMC5400653

[13] Y. Muragaki , J. Akimoto , T. Maruyama , et al., “Phase II Clinical Study on Intraoperative Photodynamic Therapy With Talaporfin Sodium and Semiconductor Laser in Patients With Malignant Brain Tumors,” Journal of Neurosurgery 119, no. 4 (2013): 845–852. 23952800 10.3171/2013.7.JNS13415

[14] T. Muroya , K. Kawasaki , Y. Suehiro , et al., “Application of PDT for Uterine Cervical Cancer,” Diagn Ther Endosc 5, no. 3 (1999): 183–190. 18493501 10.1155/DTE.5.183 PMC2362637

[15] C. Shimada , Y. Todo , H. Yamazaki , S. Minobe , and H. Kato , “Cervical Laser Vaporization for Women With Cervical Intraepithelial Neoplasia‐3,” Japanese Journal of Clinical Oncology 49, no. 5 (2019): 447–451. 30796831 10.1093/jjco/hyz001

[16] W. Suzuki , K. Ietani , T. Makabe , et al., “Prognostic Outcome of Cervical Laser Ablation Using a Holmium Yttrium‐Aluminum‐Garnet (Ho:YAG) Laser for the Treatment of Cervical Intraepithelial Neoplasia: A Single‐Center Retrospective Study,” Gynecologic Oncology Reports 53 (2024): 101405. 38757116 10.1016/j.gore.2024.101405 PMC11096838

[17] M. Mikami , M. Ikeda , H. Sato , et al., “The Use of Conization to Identify and Treat Severe Lesions Among Prediagnosed CIN1 and 2 Patients in Japan,” Journal of Gynecologic Oncology 29, no. 4 (2018): e46. 29770617 10.3802/jgo.2018.29.e46 PMC5981098

[18] H. Ichimura , S. Yamaguchi , A. Kojima , et al., “Eradication and Reinfection of Human Papillomavirus After Photodynamic Therapy for Cervical Intraepithelial Neoplasia,” International Journal of Clinical Oncology 8, no. 5 (2003): 322–325. 14586759 10.1007/s10147-003-0354-4

[19] S. Yamaguchi , H. Tsuda , M. Takemori , et al., “Photodynamic Therapy for Cervical Intraepithelial Neoplasia,” Oncology 69, no. 2 (2005): 110–116. 16118506 10.1159/000087812

[20] P. L. Martin‐Hirsch , C. Whitehurst , C. H. Buckley , J. V. Moore , and H. C. Kitchener , “Photodynamic Treatment for Lower Genital Tract Intraepithelial Neoplasia,” Lancet 351, no. 9113 (1998): 1403. 9593413 10.1016/s0140-6736(98)24019-0

[21] H. W. Wang , L. L. Zhang , F. Miao , T. Lv , X. L. Wang , and Z. Huang , “Treatment of HPV Infection‐Associated Cervical Condylomata Acuminata With 5‐Aminolevulinic Acid‐Mediated Photodynamic Therapy,” Photochemistry and Photobiology 88, no. 3 (2012): 565–569. 22150321 10.1111/j.1751-1097.2011.01060.x

[22] K. A. Keefe , Y. Tadir , B. Tromberg , et al., “Photodynamic Therapy of High‐Grade Cervical Intraepithelial Neoplasia With 5‐Aminolevulinic Acid,” Lasers in Surgery and Medicine 31, no. 4 (2002): 289–293. 12355576 10.1002/lsm.10111

[23] N. M. Inada , H. H. Buzzá , M. F. M. Leite , et al., “Long Term Effectiveness of Photodynamic Therapy for CIN Treatment,” Pharmaceuticals (Basel) 12, no. 3 (2019): 107. 31336848 10.3390/ph12030107 PMC6789515

[24] Y. P. Istomin , T. P. Lapzevich , V. N. Chalau , S. V. Shliakhtsin , and T. V. Trukhachova , “Photodynamic Therapy of Cervical Intraepithelial Neoplasia Grades II and III With Photolon,” Photodiagnosis and Photodynamic Therapy 7, no. 3 (2010): 144–151. 20728837 10.1016/j.pdpdt.2010.06.005

[25] T. Mariya , A. Nishikawa , K. Sogawa , et al., “Virological and Cytological Clearance in Laser Vaporization and Conization for Cervical Intra‐Epithelial Neoplasia Grade 3,” Journal of Obstetrics and Gynaecology Research 42, no. 12 (2016): 1808–1813. 27526956 10.1111/jog.13113

[26] K. Bodner , B. Bodner‐Adler , F. Wierrani , et al., “Cold‐Knife Conization Versus Photodynamic Therapy With Topical 5‐Aminolevulinic Acid (5‐ALA) in Cervical Intraepithelial Neoplasia (CIN) II With Associated Human Papillomavirus Infection: A Comparison of Preliminary Results,” Anticancer Research 23, no. 2c (2003): 1785–1788. 12820459

[27] P. Soergel , X. Wang , H. Stepp , H. Hertel , and P. Hillemanns , “Photodynamic Therapy of Cervical Intraepithelial Neoplasia With Hexaminolevulinate,” Lasers in Surgery and Medicine 40, no. 9 (2008): 611–615. 18951428 10.1002/lsm.20686

[28] Y. Ju and Q. Zhou , “The Impact of Photodynamic Therapy on Cellular Immune Function in Patients With Cervical HPV Infection,” Clinics 80 (2025): 100537. 39647186 10.1016/j.clinsp.2024.100537 PMC11667122

