# Supplementary material for: A Multicenter Phase II Study on Photodynamic Therapy Using Talaporfin Sodium (ME2906) and a Semiconductor Laser (PNL6405CIN) for Intraepithelial Tumors of the Cervix
Source: Reprod Med Biol. 2025 Nov 30;24(1):e70001. doi: 10.1002/rmb2.70001 (PMC12665164; doi:10.1002/rmb2.70001)
Supplement: Supplementary file 1 — Data S1: rmb270001‐sup‐0001‐supinfo.docx. [file RMB2-24-e70001-s001.docx]

**Supplementary data**

Table 1. Determination of complete response

| cervical cytology | cervical histology | comprehensive diagnosis | Treatment response |
| --- | --- | --- | --- |
| NILM | Negative | Negative | CR |
| ASC-US | Negative | LSIL |  |
|  | CIN1 |  |  |
| LSIL | Negative | LSIL |  |
|  | CIN1 |  |  |

Abbreviations: CR; Complete response, NILM; Negative for intraepithelial lesion or malignancy, ASC-US; Atypical squamous cells of undetermined significance, LSIL; Low-grade squamous intraepithelial lesion, CIN; Cervical intraepithelial neoplasia.

| Table 2. HPV clearance rate | | | | | | |
| --- | --- | --- | --- | --- | --- | --- |
|  | **Cases** | **HPV positive before PDT** | **HPV test after PDT** | | **HPV negative rate (%)** | **95% confidence interval** |
|  |  |  | **negative** | **positive** |  |  |
| **CIN3** | 70 | 67 | 55 | 12 | 82.1 | 70.8 – 90.4 |
| **CIN2** | 7 | 7 | 6 | 1 | 85.7 | 42.1 – 99.6 |
| **Total** | 77 | 74 | 61 | 13 | 82.4 | 71.8 – 90.3 |

Abbreviations: HPV; human papillomavirus, PDT; photodynamic therapy, CIN; Cervical intraepithelial neoplasia
